# High *FLT3* expression increases immune‐cell infiltration in the tumor microenvironment and correlates with prolonged disease‐free survival in patients with non‐small cell lung cancer

**DOI:** 10.1002/1878-0261.13597

**Published:** 2024-02-07

**Authors:** Łukasz Kuncman, Magdalena Orzechowska, Tomasz Milecki, Jakub Kucharz, Jacek Fijuth

**Affiliations:** ^1^ Department of Radiotherapy Medical University of Lodz Poland; ^2^ Department of External Beam Radiotherapy Nicolaus Copernicus Multidisciplinary Centre for Oncology and Traumatology Łódź Poland; ^3^ Department of Molecular Carcinogenesis Medical University of Lodz Poland; ^4^ Department of Urology Poznan University of Medical Sciences Poland; ^5^ Department of Genitourinary Oncology The Maria Sklodowska‐Curie National Research Institute of Oncology in Warsaw Poland

**Keywords:** dendritic cells, FMS‐related tyrosine kinase 3, immunotherapy, natural killer cells, radiotherapy, The Cancer Genome Atlas

## Abstract

Most of the currently used cancer immunotherapies inhibit the programmed cell death protein 1 (PD1)–programmed cell death 1 ligand 1 (PDL1) axis of T‐cells. However, dendritic cells (DCs) controlled by natural killer (NK) cells via the FMS‐related tyrosine kinase 3 (FLT3) axis are necessary for activation of T‐cells. The aim of the study was to evaluate FLT3 as a prognostic factor and determine its role in immune infiltration (with emphasis on NK cells and DCs). Using The Cancer Genome Atlas (TCGA) database, we performed bioinformatic analysis of the gene expression datasets of 501 lung squamous cell carcinoma (LUSC) and 515 lung adenocarcinoma (LUAD) patient who had corresponding clinical data [analysis was performed in R (version 4.2.0)]. Disease‐free survival (DFS) differed between the *FLT3*‐low and *FLT3*‐high expression groups, respectively, in LUSC (61.0 vs 71.3 months *P* = 0.075) and LUAD (32.7 vs 47.5 months *P* = 0.045). A tumor microenvironment (TME) with high immune infiltration and rich in T‐cell exhaustion markers was observed in the *FLT3*‐high group. We showed overexpression of NK cell and DC gene signatures in the *FLT3*‐high expression group as well as overexpression of key effector genes of the cyclic GMP‐AMP synthase (cGAS)–stimulator of interferon genes protein (STING) pathway, which is crucial in response to radiotherapy. High expression of *FLT3* in the TME was associated with immune cell infiltration (especially of NK cells and DCs), increased expression of T‐cell exhaustion markers and expression of effector genes of the cGAS‐STING pathway, which may consequently increase susceptibility to immunotherapy and radiotherapy. High *FLT3* expression correlated with prolonged DFS in the LUSC and LUAD cohorts.

## Graphical abstract

High expression of FMS‐related tyrosine kinase 3 (*FLT3*) in the non‐small cell lung cancer tumor microenvironment significantly boosts immune cell infiltration, especially by natural killer cells and dendritic cells. This enhancement may improve responses to immunotherapy and radiotherapy. FLT3 emerges as prognostic marker and disease‐free survival predictor.

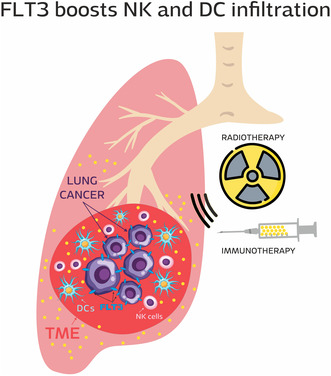

**Abbreviations**
cDC1s: conventional dendritic cells 1
cGAS: GMP–AMP synthase
CTLA4: cytotoxic T cell antigen 4
DCs: dendritic cells
FLT3: FMS‐related tyrosine kinase 3
FLT3LG: FMS‐related tyrosine kinase 3 ligand
LUAD: lung adenocarcinoma
LUSC: lung squamous cell carcinoma
MCP: microenvironment cell‐population
MCP: microenvironment cell‐population
MFA: multiple factor analysis
NK: natural killer
PD1: programmed death receptor 1
PDL1: programmed death receptor 1 ligand 1
sDCs: stimulatory dendritic cells
STING: stimulator of interferon genes
TCGA: The Cancer Genome Atlas
TME: tumor microenvironment

## 1 Introduction

Immunotherapy is one of the major breakthroughs in cancer treatment in recent years; the number of cancer types and patients treated with immunotherapy is steadily increasing [1, 2]. The most thoroughly researched and with the widest clinical use are immunotherapies based on T cells checkpoint inhibitors (programmed cell death receptor 1 (PD1/PDL1) and cytotoxic T‐lymphocyte protein 4 (CTLA4)) axes [3]. Discovery of these mechanisms was the basis for the Nobel Prize in medicine in 2018 [1]. However, despite the enormous and constantly growing possibilities, treatment with immunotherapy is effective in a moderate number of patients only in 12.46% of all treated patients in 2018, as shown in metanalysis [1]. Ineffectiveness of immunotherapy in an unfavorable scenario may result in rapid resistance or even hyperprogression [2]. Many currently conducted studies show the possibility of increasing the effectiveness of immunotherapy in influencing the immune infiltration of the tumor microenvironment other than T cells, but supporting them [2, 4, 5]. Dendritic cells (DCs) may play a significant role in this context as they prime antigen presentation and T cell activation [5, 6]. Dendritic cells (particularly conventional dendritic cells 1 (cDC1s)) are essential for effective response to immune checkpoint inhibitors (CTLA4, PD1/PDL1) that has been shown *in vivo* [7, 8].

The new perspective on control of the activity of DCs by lymphocytes, mainly natural killer cells (NK) has been published [9]. The FMS‐related tyrosine kinase 3 (FLT3), which is type III receptor tyrosine kinase expressed exclusively on hematopoietic stem cells and DCs, is considered to be crucial in FLT3 ligand (FLT3LG)‐mediated control of dendritic cells by NK cells [9, 10, 11]. For an illustrative example, genetic and cellular ablation of NK cells in melanoma mice turned off FLT3‐mediated control and NK cells did not form conjugates with stimulatory dendritic cells (BDCA3+) (sDCs) [9]. Preclinical data also indicate a key role of NK‐FLT3/FLT3LG‐DC axis in the efficacy of the radio‐immunotherapy combination [12]. The activation of NK cells within the tumor microenvironment (TME) was shown to be viable strategy for NK cell‐based immunotherapy to counteract resistance in T cells deficient HPV‐negative orthotopic models of head and neck squamous cell carcinomas [12]. The addition of FLT3L to radiotherapy and anti‐CD25 treatment significantly diminished MOC2 buccal tumors [12]. Radiotherapy damaging cancer cells induces antitumor adaptive immunity by releasing tumor antigens, RNA, DNA to cytoplasm [13]. Conventional dendritic cells 1 uniquely respond to those signals making radiotherapy a natural candidate for combination with dendritic cell‐based immunotherapy and in view of above‐mentioned axis also for NK cells [13, 14].

The cyclic GMP–AMP synthase (cGAS)–stimulator of interferon genes (STING) pathway is particularly interesting in the context of the present work as it is one of most promising pathways in the context of immunomodulating anti‐cancer activity. The cGAS‐STING pathway signaling is activated in response to dsDNA that is present as a result of the action of ionizing radiation and triggers dendritic cells [15, 16]. The cGAS‐STING is believed to be crucial in radiation‐induced DNA damage immune response [17].

The FLT3/FLT3L axis, widely recognized for its role in hematopoiesis, has yet to be clearly established in the context of TME regulation [18]. As shown above, this perspective is evolving, as recent preclinical models reveal that the FLT3/FLT3L axis may also play a significant role in modulating responses to radiotherapy and immunotherapy [12]. It is particularly important to explore these possibilities in the context of lung cancer, where chemoradiation with consolidating immunotherapy is the standard of care in stage III, and trials are ongoing regarding other indications [19].

Here we show the relationship between *FLT3* gene expression and disease‐free survival in patients with lung squamous cell carcinoma (LUSC) and lung adenocarcinoma (LUAD) and immune cell infiltration with particular emphasis on the role of NK and stimulatory DC cells subpopulations. We present relationship between the *FLT3* and the expression of cGAS‐STING pathway genes pivotal in the activation of dendritic cells and response to radiotherapy [15, 20].

## 2 Materials and methods

The lung squamous cell carcinoma (LUSC) and lung adenocarcinoma (LUAD) gene expression data sets were obtained from The Cancer Genome Atlas (TCGA) repositories through GDAC Firehose (RNAseqV2, RSEM normalized, data status of 28th Jan 2016) including 501 and 515 patients, respectively, with corresponding clinical data [21]. The method of biospecimen procurement, mRNA isolation and sequencing in TCGA cohort has been previously described [22, 23]. All patients provided written informed consent to conduct genomic studies in accordance with local Institutional Review Boards. Only patients with expression and Disease‐Free Survival (DFS) data were included into further analysis. Spearman's rank correlation test was used to correlate the relationship between *FLT3* and *FLT3LG* expression.

Evaluate Cutpoints RShiny application through the maxstat algorithm for R environment was used to evaluate correlation of *FLT3* gene expression and DFS according to defined optimal cutpoint [24]. Variable such as “patient.person_neoplasm_cancer_status” was regarded as event and “patient.days_to_last_followup” as time of observation for DFS. Subsequently, patients were stratified into subgroups of *FLT3*‐low and *FLT3*‐high expression according to defined cutpoint (i.e. expression below and above the determined cutpoint, respectively).

Microenvironment cell‐population (MCP)‐counter method was used for quantification of immune infiltration through deconvolution of transcriptomic data among *FLT3*‐low and *FLT3*‐high groups [25]. The analysis was extended with the association of *FLT3*‐low/*FLT3*‐high with infiltration by sDCs (BDCA3+) and NK cells. As a surrogate for sDCs and NK cells abundance, expression of previously described signature genes of sDCs (*KIT*, *CCR7*, *BATF3*, *FLT3* (excluded from MCPcounter analysis), *ZBTB46*, *IRF8*, *BTLA*, *MYCL1*) and NK (*GNLY*, *KLRC3*, *FLT3LG KLRD1*, *KLRF1*, *NCR1*) has been adopted [9, 26, 27]. Spatial partitioning of LUSC and LUAD patients regarding their clinical characteristics was performed based on models of *FLT3*‐low/*FLT3*‐high and T‐cell exhaustion marker gene expression (*PDCD1* (PD1), *CD274* (PDL1), *PDCD1LG2* (PDL2), *CTLA4*, *LAG3*, *HAVCR2* (TIM3), *GZMB*, *BTLA*, *CD160*, *CD244* (2B4), *TIGIT*) through the Multiple Factor Analysis (MFA) being an extension of the Principal Component Analysis (PCA) allowing to mix variables of different types [28, 29, 30, 31].

The cGAS‐STING pathway genes such as *C6orf150* (CGAS), *DDX41*, *DTX4*, *IFI16*, *IRF3*, *MRE11A*, *NLRC3*, *NLRP4*, *PRKDC*, *STAT6*, *TMEM173* (STING1), *TBK1*, *TREX1*, *TRIM21*, *XRCC5*, *XRCC6* were retrieved from Molecular Signatures Database (MSigDB) and analyzed based on Reactome Pathways database [32, 33, 34]. Models explaining association of *FLT3*‐low and *FLT3*‐high with cGAS‐STING pathway genes expression that differentiate LUSC and LUAD patients were performed with MFA. Commonly used gene aliases have been placed in parentheses.

All analyses were performed within r v.4.2.0 environment and packages such as factominer, factoextrar, survival, and immunedeconv [35]. The entire bioinformatics analysis code in the r environment and the database obtained and processed is available at: https://1drv.ms/f/s!AqwHYmZlPESTgo11GL6ZpFpmNnK5Uw?e=ReYm1G.

## 3 Results

### 3.1 Clinical characteristics of cohort

Five hundred and fifteen patients (277 women, 238 men) with lung adenocarcinoma and 501 patients (130 women, 371 men) with lung squamous cell carcinoma with expression and data enabling evaluation of disease‐free survival (DFS) data were included in the analysis. Median age was 66 (38–88) in LUAD and 68 (39–90) in LUSC. In LUAD 426 patients had smoking history, 512 were previously untreated, 3 had neoadjuvant treatment. In LUSC 471 patients had smoking history, 494 were previously untreated, 7 had neoadjuvant treatment. Patients were predominantly in localized stage; 397 out of 515 in LUAD (stage I‐275, stage II‐122, stage III‐84, stage IV‐26), 406 out of 501 in LUSC (stage I‐244, stage II‐162, stage III‐84, stage IV‐7). Only clinical stage from above‐mentioned clinical features was associated with DFS (Fig. S1).

### 3.2 FLT3 as a prognostic factor

Gene expression data and variables such as “patient.person_neoplasm_cancer_status”, “patient.days_to_last_followup” data were available for 422 LUAD and 392 LUSC patients and those individuals were included into further analysis. *FLT3* expression was moderately correlated with *FLT3‐L* expression in both cases (LUSC: rho = 0.52, *P* < 0.001, LUAD: rho = 0.48, *P* < 0.001) (Fig. S2).

**Fig. 1:**
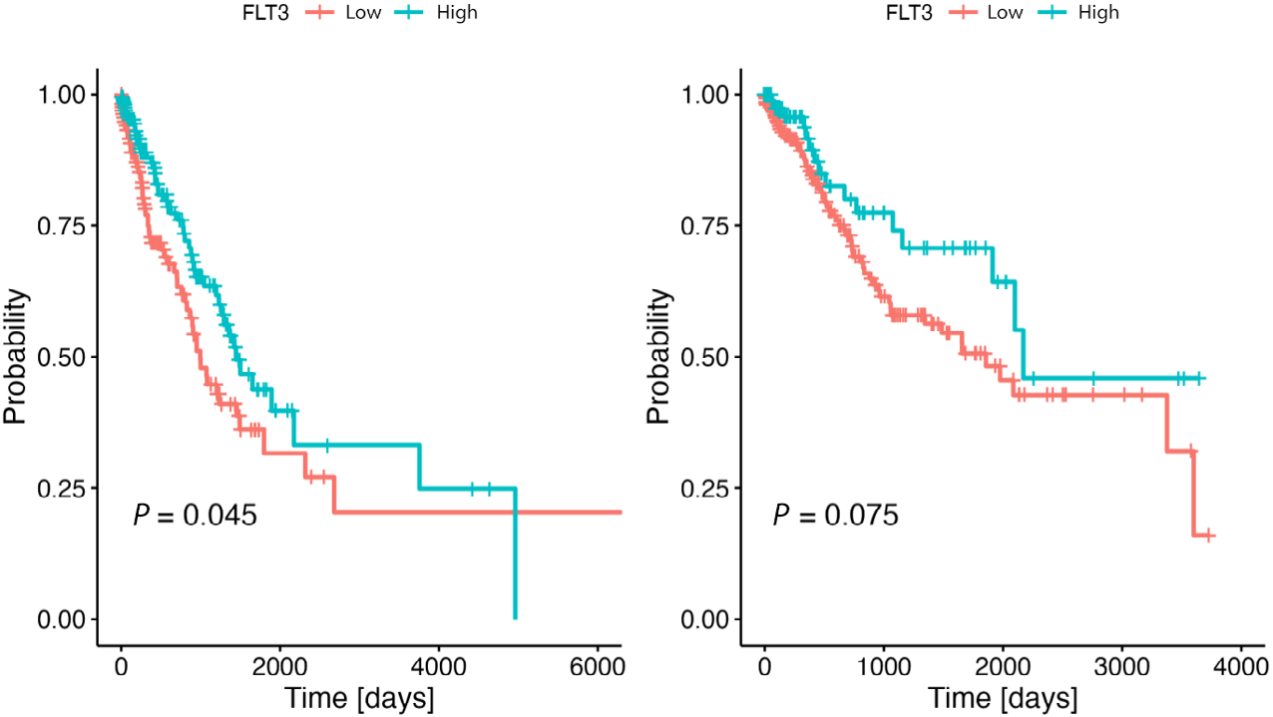
Disease‐free survival of the patients with lung adenocarcinoma (LUAD) and lung squamous cell carcinoma (LUSC) according to FMS‐related tyrosine kinase 3 (FLT3)‐low and FLT3‐high gene expression group in tumor microenvironment. DFS for LUAD cohort is shown on left, DFS for LUSC cohort is shown on right. Cutpoints RShiny application through the maxstat algorithm for R environment was used for calculations.

The dichotomization into *FLT3*‐low/*FLT3*‐high groups was performed according to DFS. Optimal cutpoint was determined as 14.51 for LUSC and 11.31 for LUAD. Disease‐free survival differed between the *FLT3*‐low and *FLT3*‐high group, respectively, for both LUSC (61.0 vs 71.3 months HR = 0.61, 95% CI: 0.35–1.06, *P* = 0.075) and LUAD (32.7 vs 47.5 months HR = 0.69, 95% CI: 047–0.99, *P* = 0.045) (Fig. 1).

### 3.3 FLT3 expression and immune infiltration

**Fig. 2:**
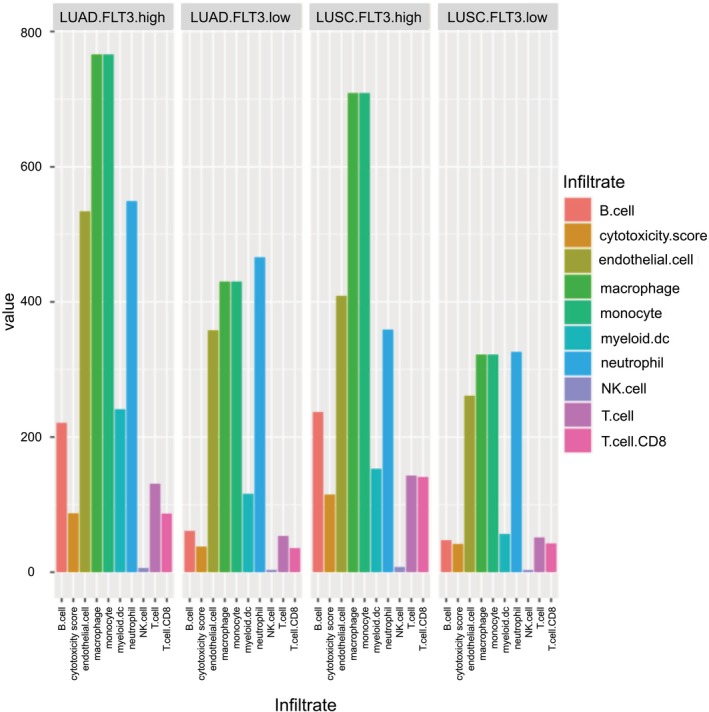
Immune cell infiltration assessed using microenvironment cell‐population according to FMS‐related tyrosine kinase 3 (FLT3)‐low and FLT3‐high gene expression group in tumor microenvironment of the patients with lung adenocarcinoma (LUAD) and lung squamous cell carcinoma (LUSC). The figure shows comparison of abundance of various immune cells between FLT3‐low and FLT3‐high gene expression group in tumor microenvironment within two different types of lung cancer (LUAD and LUSC). The immune cells are itemized on the right, with each type corresponding to the color of the bars displayed in the chart.

The results of the Microenvironment Cell‐Population (MCP)‐counter method for the LUSC and LUAD cohorts show differences in the relative abundance of various cell populations in the tumor microenvironment of the two types of lung cancer according (Table S1, Fig. 2). In both the LUSC and LUAD cohorts, the *FLT3*‐high expression group had higher infiltration of all assessed immune cells (T cells, T cells CD8+, cytotoxic score, NK cells, B cells, monocytes, macrophages, myeloid dendritic cells, neutrophils, endothelial cells) and cancer‐associated fibroblasts compared to the *FLT3*‐low expression group (Table S1, Fig. 2). NK cells were the rarest in tumor microenvironment infiltration but significantly more common in the *FLT3*‐high group; value – 3.28, 3.46 for the *FLT3*‐low group and 7.74, 6.20 for the *FLT3*‐high group for LUSC and LUAD, respectively. sDCs infiltrated the microenvironment in greater numbers similar to NK to a much greater extent in the *FLT3*‐high group; value: 56.68, 115.57 for the *FLT3*‐low group and 152.75, 241.27 for the *FLT3*‐high group for LUSC and LUAD, respectively.

**Fig. 3:**
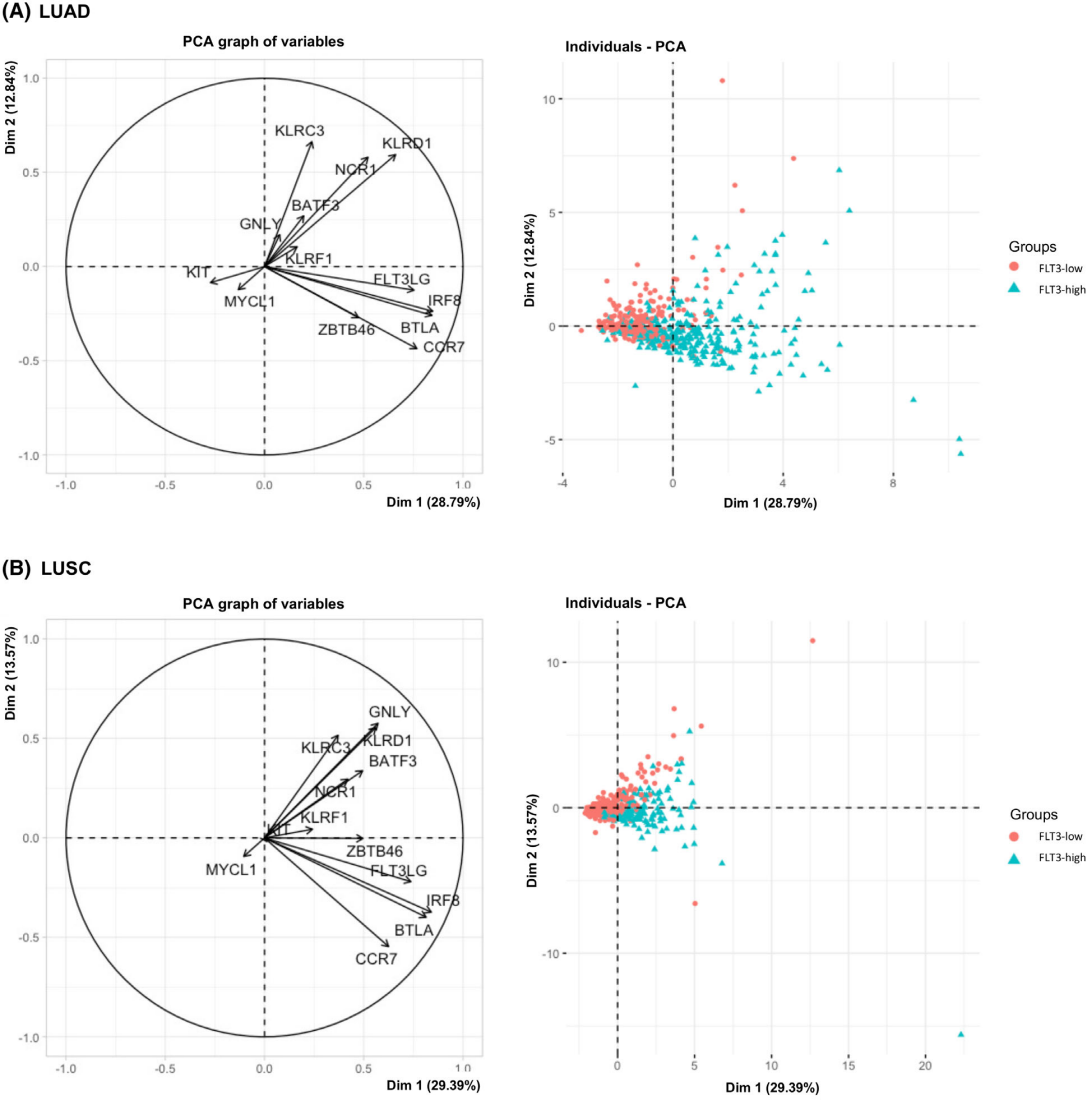
Multiple factor analysis (MFA) of natural killer (NK) cells 1 and stimulatory dendritic cells (DCs) gene signature expression and FMS‐related tyrosine kinase 3 (FLT3)‐low/FLT3‐high gene expression group in tumor microenvironment of the patients with lung adenocarcinoma (LUAD) and lung squamous cell carcinoma (LUSC). A correlation circle demonstrates the relationship between the expression of signature genes of NK cells 1 and stimulatory DCs (left) and the spatial distribution of individuals with low and high FLT3 gene expression (right). These patterns are depicted for LUAD (A) at the top and LUSC (B) at the bottom of the figure.

To confirm above findings gene signature of natural killers cells (*GNLY*, *KLRC3*, *FLT3LG*, *KLRD1*, *KLRF1*, *NCR1*) and stimulatory dendritic cells (*KIT*, *CCR7*, *BATF3*, *ZBTB46*, *IRF8*, *BTLA*, *MYCL1*) was derived from previously published research and used for partitioning of LUAD and LUSC patients according to their *FLT3*‐NK1‐sDC gene expression patterns through MFA [9, 26, 27]. The model revealed surprisingly significant spatial grouping of patients according to *FLT3* expression group (Fig. 3). Our model explained large proportion of the variation in the data for LUAD (dimension 1–2 41.64%) and for LUSC (dimension 1–2 42.96%) pointing the strong correlation between *FLT3* expression with expression of NK cells and sDCs gene signature as shown by correlation circle and individual factor map. *FLT3*‐high expression group had higher infiltration of both natural killer cells and stimulatory dendritic cells. Lowered *FLT3* expression correlated with increase in *MYCL1*, and additionally *KIT* in LUSC (Fig. 3).

### 3.4 Correlation of FLT3 with T‐cell exhaustion markers

**Fig. 4:**
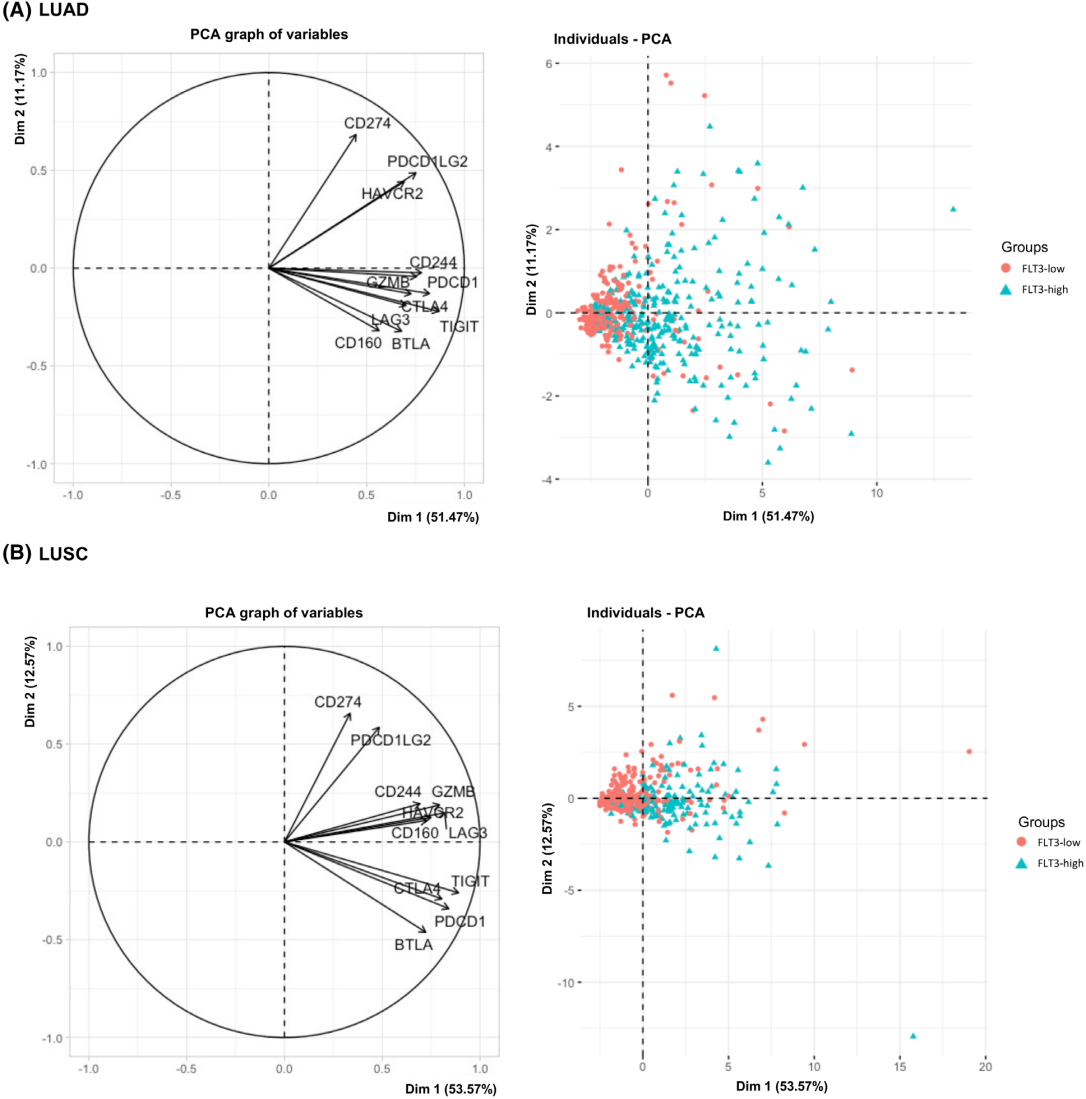
Multiple factor analysis (MFA) of T‐cell exhaustion gene signature expression and FMS‐related tyrosine kinase 3 (FLT3)‐low/FLT3‐high gene expression group in tumor microenvironment of the patients with lung adenocarcinoma (LUAD) and lung squamous cell carcinoma (LUSC). A correlation circle demonstrates the relationship between the T‐cell exhaustion gene signature (left) and the spatial distribution of individuals with low and high FLT3 gene expression (right). These patterns are depicted for LUAD (A) at the top and LUSC (B) at the bottom of the figure.

We derived T‐cell exhaustion marker genes (PDCD1 (PD1), CD274 (PDL1), PDCD1LG2 (PDL2), CTLA4, LAG3, HAVCR2 (TIM3), GZMB, BTLA, CD160, CD244 (2B4), TIGIT) from previously published papers and analyzed above‐mentioned gene expression according to FLT3‐low/FLT3‐high expression group [28]. Our model successfully accounted for a meaningful amount of the variability in dimensions 1 and 2 in the LUAD dataset (62.64%) and the LUSC dataset (66.14%). These data indicate a strong association between FLT3 expression and T‐cell exhaustion markers, as demonstrated by the correlation circle and individual factor map (Fig. 4) for both LUAD and LUSC. Individuals from *FLT3‐high* group had high expression of all accessed T‐cell exhaustion marker genes.

### 3.5 Role of *cGAS‐STING* pathway in *NK‐FLT3‐sDC*

The cGAS‐STING pathway genes (*C6orf150* (cGAS), *DDX41*, *DTX4*, *IFI16*, *IRF3*, *MRE11A*, *NLRC3*, *NLRP4*, *PRKDC*, *STAT6*, *TMEM173* (STING1), *TBK1*, *TREX1*, *TRIM21*, *XRCC5*, *XRCC6*) were used to associate cGAS‐STING pathway involvement in *FLT3‐FLT3LG* signaling. The members of the cGAS‐STING pathway were divided into subgroups according to convergent branch of the pathway based on Reactome analysis [32].

**Fig. 5:**
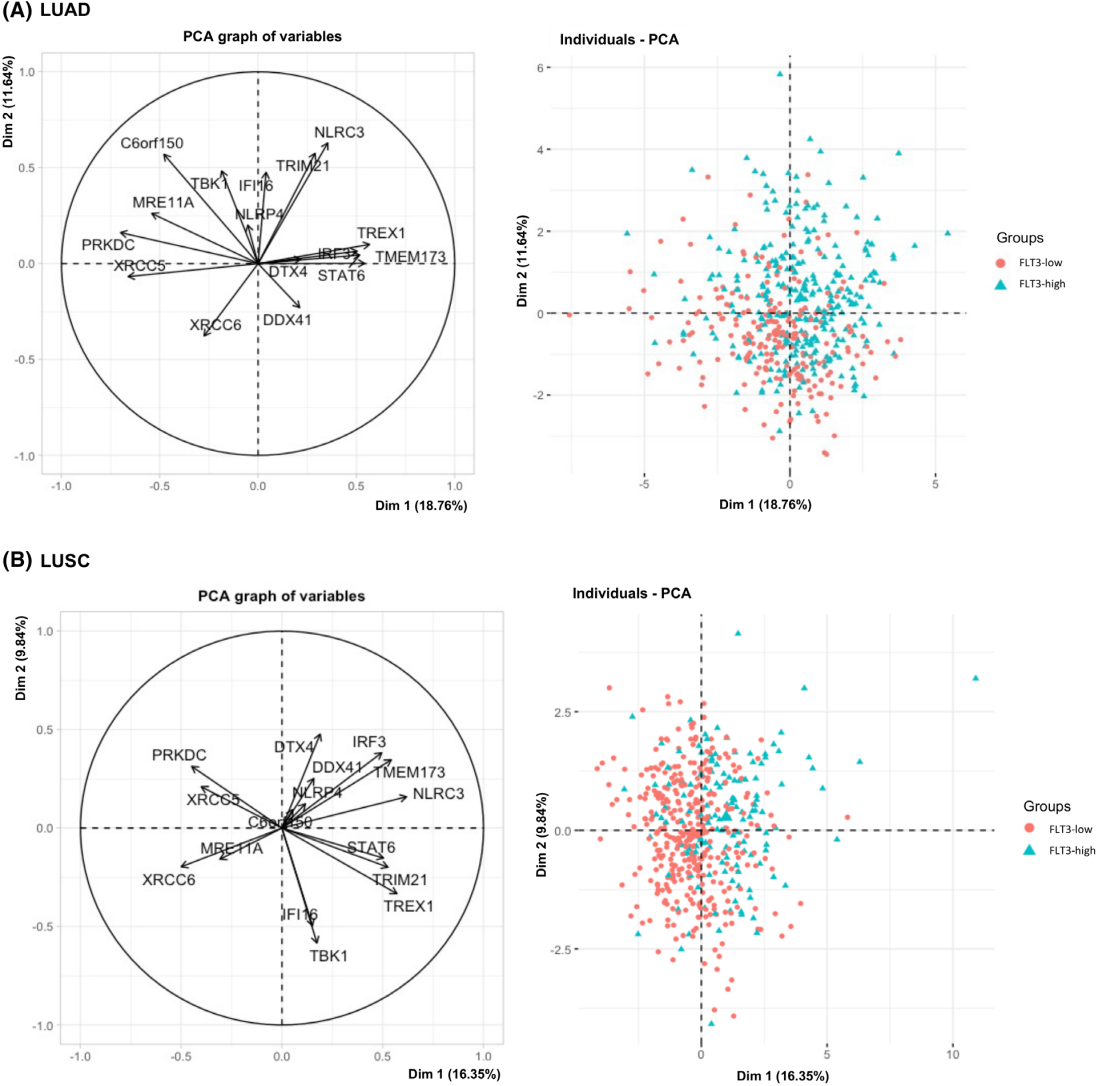
Multiple factor analysis (MFA) of the cyclic GMP–AMP synthase (cGAS)–stimulator of interferon genes (STING) pathway gene expression and FMS‐related tyrosine kinase 3 (FLT3)‐low/FLT3‐high gene expression group in tumor microenvironment of the patients with lung adenocarcinoma (LUAD) and lung squamous cell carcinoma (LUSC). A correlation circle demonstrates the relationship between the cGAS‐STING genes (left) and the spatial distribution of individuals with low and high FLT3 gene expression (right). These patterns are depicted for LUAD (A) at the top and LUSC (B) at the bottom of the figure.

According to the correlation circle (Fig. 5A,B) *XRCC5*, *XRCC6* and *PRKDC* expression for both LUAD and LUSC, *C6orf150* (cGAS) for LUSC were corelated predominantly in *FLT3*‐low group.

Genes such as *TRIM21*, *DDX41*, *TREX1*, *NLRC3*, *STAT6*, *TBK1*, *IRF3*, *TMEM173* (STING1), *DTX4*, *NLRP4* corresponded with the position of the individuals with *FLT*‐high both for LUAD and LUSC (Fig. 5A,B). According to the above analyses, increased expression of *FLT3* was associated with the expression of *TRIM21/DDX1*, *TREX1* as initial messengers stimulating STING signaling but not with *C6orf150* (cGAS) or *Ku70:80* dimer (XRCC5, XRCC6)/*PRKDC* patterns. High expression of NLRC3, STAT6, TBK1, IRF3, TMEM173 (STING1), DTX4, genes constituting the central part of the cGAS‐STING (Fig. S3) analysis was present in the FLT3‐high group. All above‐mentioned genes, except for NLRC3, has cGAS‐STING stimulating function.

## 4 Discussion

In our study, we show that *FLT3* expression is a favorable prognostic factor in patients with lung squamous cell carcinoma and lung adenocarcinoma. Those with higher expression of *FLT3* (i.e. above the cutpoint) had prolonged disease‐free survival (DFS). To the best of our knowledge, so far there are no other studies investigating the role of *FLT3* as prognostic factor in patients with lung cancer. Recently published data from patients with breast (*FLT3*) and cervical cancer (*FLT3LG*) stay in line with our results [29, 36].

Subsequently, we tried to explain the mechanisms responsible for the relationship of *FLT3* and DFS. *FLT3/FLT3LG* is involved in hematopoiesis and natural killer cell‐mediated control of dendritic cells [5, 9]. We observed a strong association of high *FLT3* expression with immune cell infiltration, which was also observed in the cervical and breast cancer studies [29, 36]. Immune cell infiltration (especially cytotoxic CD8+ lymphocytes) is a recognized prognostic factor in lung cancer, which may partially explain the beneficial effect of *FLT3* expression on DFS [37, 38]. Moreover, our analysis revealed strong correlation of *FLT3* expression and T‐cell exhaustion markers genes (as a brief recap, MFA explained 62% and 66% of data variation in LUAD and LUSC, respectively). TME with high immune infiltration and rich in T‐cell exhaustion markers, which was observed in *FLT3*‐high group is prone to positive effect of immunotherapy [9].

We extended our analysis with an evaluation of the genetic signature of natural killer cells and dendritic cells. The MFA revealed clear spatial partitioning of patients; individuals with high *FLT3* expression had heightened expression of marker genes of DCs and NK cells. NK cells activate DCs via the FLT3/FLT3LG signaling pathway [5]. DC's in turn prime cytotoxic CD8 + lymphocytes, which are pivotal in mediating anti‐cancer immunity [38]. FLT3/FLT3LG axis may be important player in this cascade. Recently published report stressed out the key role of NK cells in radiotherapy‐driven immune response [39]. NK infiltration is low in NSCLC (similarly to other malignancies) patients and associated with better survival if present in greater extend [40, 41]. In view of above analysis, *FLT3* expression plays as marker of NK/DC infiltration. In our previous study, we demonstrated increased blood levels of *FLT3LG* during chemoradiotherapy and discussed the possible implications for combining radiotherapy with immunotherapy [42]. CXCL8/IL‐8–dependent mechanism of radiation‐induced chemoattraction of NK was described recently, FLT3/FLT3LG axis may act similarly in view of our previous reports.

We observed high expression of key effector genes of the cGAS‐STING pathway in *FLT3*‐high expression group, despite increased expression of TRIM21 and TREX known as inhibitors of cGAS‐STING initiation. Of the initial tracks, only the DDX41 overexpression was present in individuals with *FLT3‐*high in our MFA. Both *TREX1* and *TRIM21* are of interest in the context of the immune effect induced by radiation therapy. *TRIM21* has the ability to regulate the degradation of *Oct‐1*, which in turn makes cancer stem cells more responsive to chemoradiation treatment [43]. Additionally, the activation of *TRIM21* by Dihydroartemisinin (*DHA*) was discovered to modulate EMT‐related proteins by blocking PDL1, thereby increasing the sensitivity of non‐small‐cell lung cancer to radiation therapy [44]. TREX1, which is activated by radiation doses higher than 12–18 Gray, degrade cytosolic DNA accumulated as a result of radiation and in turn diminishes the immunogenicity [45].

While the basic concept of FLT3L‐induced NK cells, DCs expansion and hematopoiesis is established, our work contributes to the field by exploring these mechanisms in tumor microenvironment of NSCLC cohort of patients what can be crucial in response to treatment as describe above [11]. Analogically it has been shown for currently used PD1/PDL1 immunotherapies [46]. Preclinical studies have highlighted, as well, the importance of the NK‐FLT3/FLT3LG‐DC axis in the efficacy of combined radio‐immunotherapy, as demonstrated by Bickett et al [12]. FLT3LG concentration in blood is elevated during chemoradiotherapy what we presented recently [42]. This adds significance to current study. The major limitation of our study is that it is a quantitative, not qualitative, data analysis and lacks in validation due to unavailability of appropriate independent cohorts. Nevertheless, our study was performed including a total of 1016 NSCLC TCGA patients. Additionally, MFA which we used could identify underlying patterns or factors that may not be apparent from a simple analysis of individual variables or groups. Our results derived thus a hypothesis; more in‐depth conclusions can be drawn from prospective single‐cell function evaluation that we plan as next step.

## 5 Conclusion

In conclusion, our study, the first of its kind, analyzes genomic material from 1016 NSCLC cases and shows the effect of high expression of *FLT3* on tumor microenvironment. Notably, high *FLT3* expression arouses as positive prognostic factor for disease‐free survival. We demonstrated a significant association of FLT3 expression and immune cell infiltration (especially NK cells and DCs), T‐cell exhaustion markers, effector genes of the cGAS‐STING pathway. This gives valuable insights into sensitivity of NSCLC to immunotherapy and radiotherapy. Our research may contribute to the design of new therapies based on NK‐DCs. Other studies provide evidence for combining radiotherapy with DC‐NK‐targeted therapies [39]. Additionally, first clinical trials show the activity of *FLT3LG* in combination with stereotactic radiotherapy in non‐small cell lung cancer [47]. Last but not least, the *FLT3*‐based treatment is well researched and widely used in FLT3‐mutated acute myeloid leukemia, which may facilitate simple application to solid tumors [48].

## Conflict of interest

ŁK, MO, TM, JK, and JF disclose any actual or potential conflict of interest including any financial, personal or other relationships with other people or organizations within 3 years of beginning the submitted work that could inappropriately influence, or be perceived to influence, their work.

## Author contributions

ŁK contributed to conceptualization, methodology, validation, investigation, writing—original draft preparation, visualization, supervision, and funding acquisition. MO contributed to methodology, software, validation, and visualization. TM and JK contributed to writing—review and editing. JF contributed to validation, supervision, and funding acquisition. All authors have read and agreed to the published version of the manuscript.

### Peer review

The peer review history for this article is available at https://www.webofscience.com/api/gateway/wos/peer‐review/10.1002/1878‐0261.13597.

## Supporting information


**Fig. S1.** Disease‐free survival (DFS) in lung adenocarcinoma (LUAD) and lung squamous cell carcinoma (LUSC) and clinical data.
**Fig. S2.** Correlation of FMS‐Related Tyrosine Kinase 3 (FLT3) with FMS‐Related Tyrosine Kinase 3 Ligand (FLT3LG).
**Table S1.** Microenvironment Cell Population in Lung Squamous Cell Carcinoma (LUSC) and Lung Adenocarcinoma (LUAD) According to FMS‐Related Tyrosine Kinase 3 (FLT3) Expression.

## Data Availability

The entire bioinformatics analysis code in the R environment and the database obtained and processed is available at: https://1drv.ms/f/s!AqwHYmZlPESTgo11GL6ZpFpmNnK5Uw?e=ReYm1G.
