# Supplementary material for: High FLT3 expression increases immune‐cell infiltration in the tumor microenvironment and correlates with prolonged disease‐free survival in patients with non‐small cell lung cancer
Source: Mol Oncol. 2024 Feb 7;18(5):1316–26. doi: 10.1002/1878-0261.13597 (PMC11076988; doi:10.1002/1878-0261.13597)

Supplementary materials

Figure S1

Disease-free survival (DFS) in lung adenocarcinoma (LUAD) and lung squamous cell carcinoma (LUSC) and clinical data. Multivariate regression forest plot of DFS and clinical data.


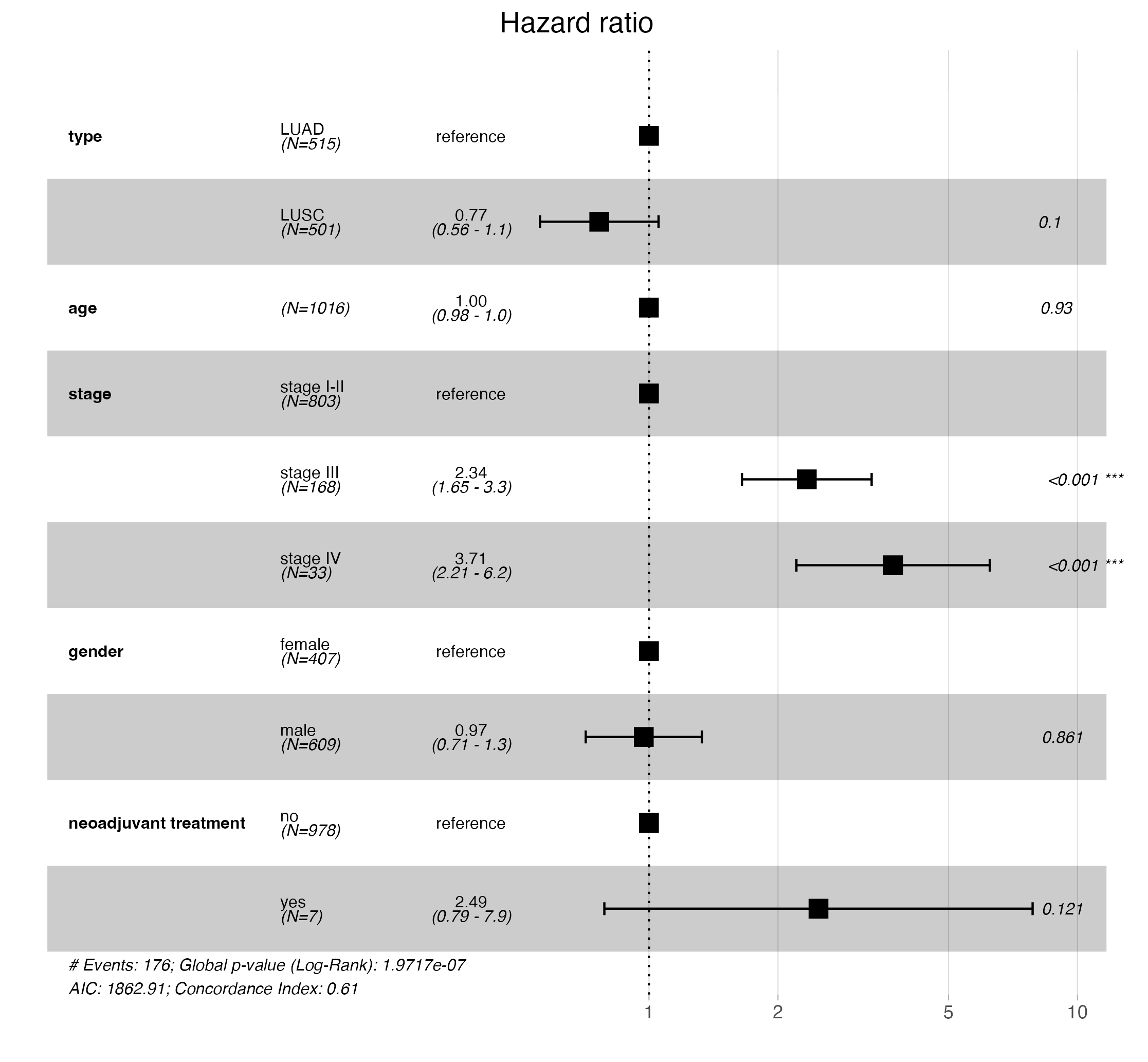


Figure S2

Correlation of FMS-Related Tyrosine Kinase 3 (FLT3) with FMS-Related Tyrosine Kinase 3 Ligand (FLT3LG). A) Lung Squamous Cell Carcinoma (LUSC) and B) Lung Adenocarcinoma (LUAD) - The Cancer Genome Atlas (TCGA) Cohorts


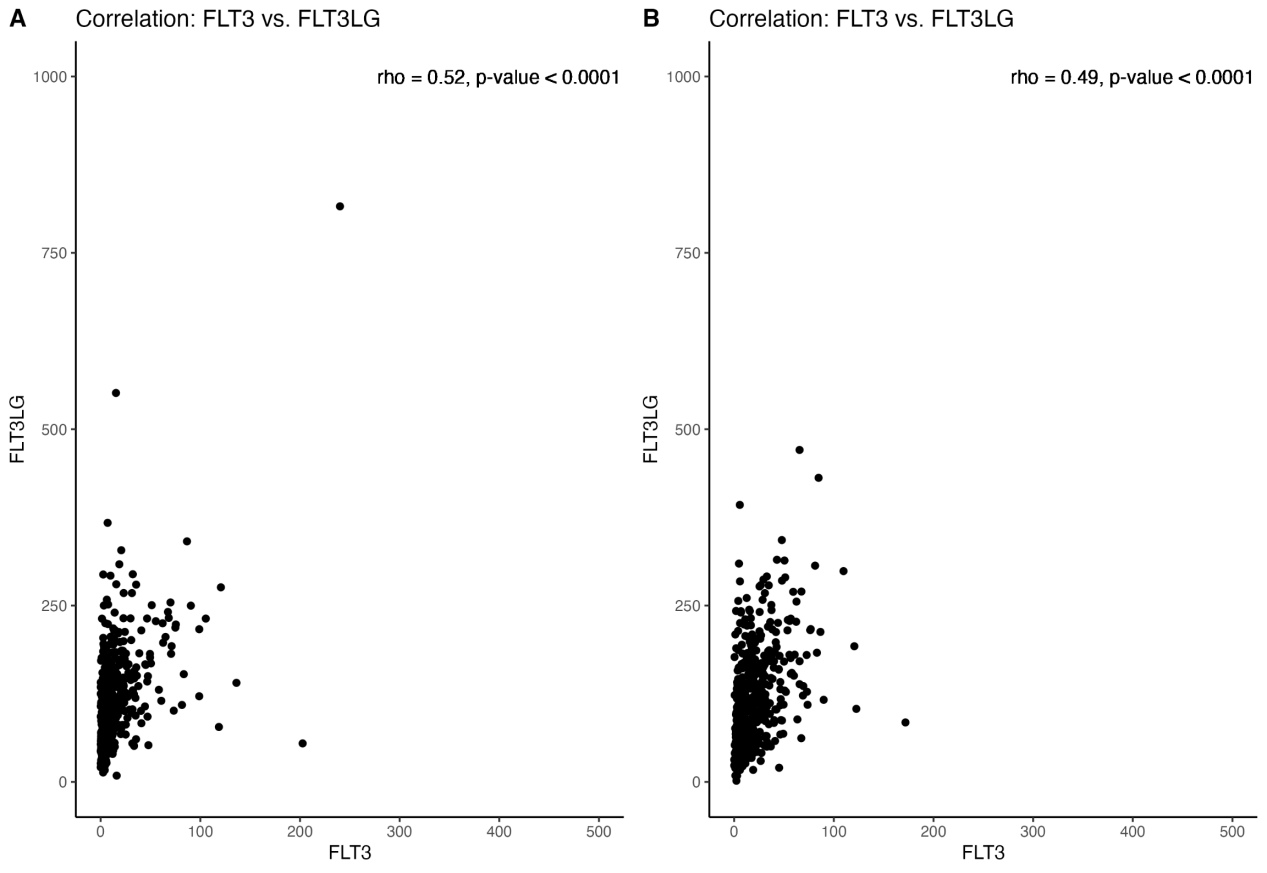


Table S1:

Microenvironment Cell Population in Lung Squamous Cell Carcinoma (LUSC) and Lung Adenocarcinoma (LUAD) According to FMS-Related Tyrosine Kinase 3 (FLT3) Expression.
The data are presented in a tabular format for detailed analysis.

|  | LUSC cohort | | | LUAD cohort | | | |
| --- | --- | --- | --- | --- | --- | --- | --- |
| Infiltrating cells type | FLT3-low expression group | FLT3-high expression group | Realtive diffrence | FLT3-low expression group | FLT3-high expression group | Realtive diffrence |  |
| T cell | 51.42 | 142.83 | 278% | 53.62 | 131.38 | 245% |  |
| T cell CD8+ | 42.51 | 141.47 | 333% | 35.58 | 86.89 | 244% |  |
| cytotoxicity score | 41.68 | 114.69 | 275% | 37.79 | 87.31 | 231% |  |
| NK cell | 3.28 | 7.74 | 236% | 3.46 | 6.20 | 179% |  |
| B cell | 47.25 | 236.52 | 501% | 61.38 | 220.50 | 359% |  |
| Monocyte | 322.10 | 709.11 | 220% | 430.30 | 766.23 | 178% |  |
| Macrophage/Monocyte | 322.10 | 709.11 | 220% | 430.30 | 766.23 | 178% |  |
| Myeloid dendritic cell | 56.68 | 152.75 | 270% | 115.57 | 241.27 | 209% |  |
| Neutrophil | 325.84 | 358.55 | 110% | 466.37 | 549.18 | 118% |  |
| Endothelial cell | 261.24 | 409.03 | 157% | 357.80 | 533.85 | 149% |  |
| Cancer associated fibroblast | 11701.36 | 22721.27 | 194% | 10457.12 | 12452.60 | 119% |  |

Figure S3

Reactome gene pathway diagram according to: <https://reactome.org/PathwayBrowser/#/R-HSA-1834949&SEL=R-HSA-976051&PATH=R-HSA-168256,R-HSA-168249>


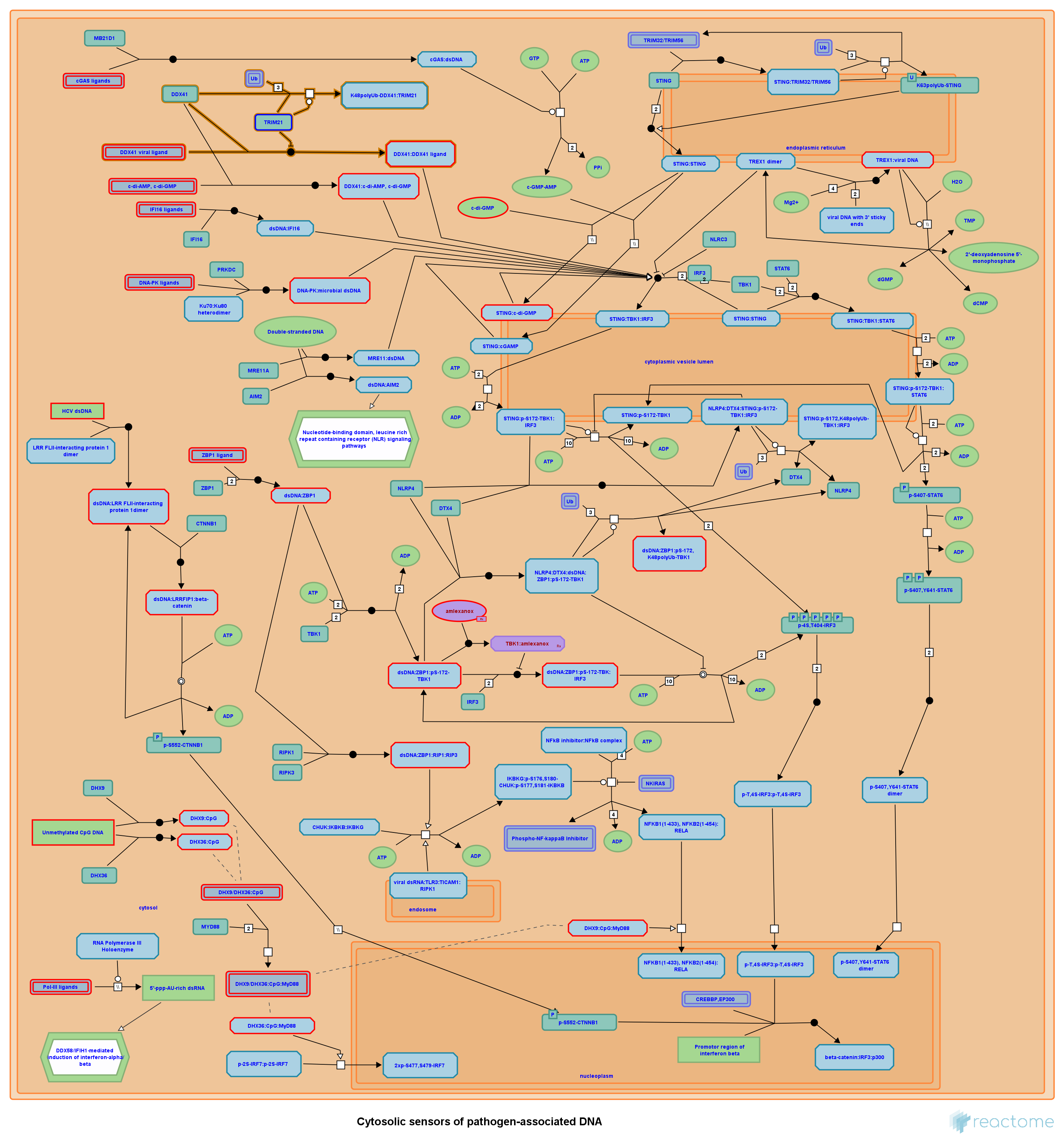

Supplement: Supplementary file 1 — Fig. S1. Disease‐free survival (DFS) in lung adenocarcinoma (LUAD) and lung squamous cell carcinoma (LUSC) and clinical data. Fig. S2. Correlation of FMS‐Related Tyrosine Kinase 3 (FLT3) with FMS‐Related Tyrosine Kinase 3 Ligand (FLT3LG). Table S1. Microenvironment Cell Population in Lung Squamous Cell Carcinoma (LUSC) and Lung Adenocarcinoma (LUAD) According to FMS‐Related Tyrosine Kinase 3 (FLT3) Expression. [file MOL2-18-1316-s001.docx]
